# Unmasking Postural Hypotension: A Stroke Mimic in an Elderly Patient

**DOI:** 10.7759/cureus.88337

**Published:** 2025-07-19

**Authors:** Zhi Tian Chen, Lih Yin Chong, Rizuan Mohamed, Deepika Gorthy

**Affiliations:** 1 Department of Medicine for Older People, Mid and South Essex NHS Foundation, Basildon, GBR; 2 Acute Medical Unit, Mid and South Essex NHS Foundation, Basildon, GBR

**Keywords:** adult and geriatric clinical medicine, elderly patients, orthostatic cerebral hypoperfusion syndrome, postural orthostatic hypotension, stroke mimic

## Abstract

Postural hypotension (also known as orthostatic hypotension) is a common but often underrecognized condition, particularly prevalent among the elderly. Despite its frequency, its clinical significance is sometimes underestimated in routine clinical practice. Failure to recognize this condition can lead to repeated hospitalizations, unnecessary treatments, and increased healthcare costs. We report the case of a 79-year-old male who presented to us with multiple stroke-like episodes in the past six months. He underwent multiple CT scans of the brain/neck and MRI head scans. However, these investigations did not reveal any acute ischemic or hemorrhagic changes or any other acute intracranial abnormality. A simple and non-invasive lying/standing blood pressure measurement done in this admission managed to uncover the mystery of stroke mimic. This case illustrates the value of taking into account postural hypotension in the differential diagnosis of transient neurological symptoms.

## Introduction

Postural hypotension (also known as orthostatic hypotension) is a common but often underrecognized condition, with an estimated overall prevalence of up to 20%. Among various inpatient series, the prevalence of orthostatic hypotension in older adult patients is as high as 60% [1]. It is defined as a fall in systolic blood pressure of at least 20 mmHg (at least 30 mmHg in patients with hypertension) and/or a fall in diastolic blood pressure of at least 10 mmHg within 3 minutes of standing [2]. Orthostatic hypotension can be symptomatic or asymptomatic. Symptoms typically occur in response to changing upright posture or may also occur after eating, heat exposure, exertion, or prolonged standing [1]. Despite its frequency, we often underestimate its clinical significance in routine practice. Failure to recognize this condition can lead to repeated hospitalizations, unnecessary treatments, and increased healthcare costs [3]. We present a case of a 79-year-old male who presented to us with symptomatic postural hypotension, disguised under recurrent episodes of stroke-like episodes [4].

## Case presentation

A 79-year-old man presented to our Emergency Department (ED) with slurred speech and confusion. According to his wife, his symptoms started suddenly at 16:00 after he returned from walking his dog. He was initially assessed by paramedics at 16:50. They found him to be confused, with a Glasgow Coma Scale (GCS) of 14/15 and dysphasic. No other focal neurological deficits were identified. His vital signs were stable, and he was subsequently transferred to the ED.

He had a medical history of multiple transient ischemic attacks, atrial fibrillation, benign prostatic hyperplasia, hypercholesterolemia, ischemic heart disease, hypertension, and type 2 diabetes mellitus. He had six recent hospitalizations in the past six months for similar episodes; he had received thrombolysis in August and November of the previous year for suspected strokes at our hospital. He was then discharged well without any major complications. His home medications include atorvastatin, clopidogrel, co-codamol, isosorbide mononitrate, lansoprazole, metformin, tamsulosin, and rivaroxaban.

Upon arrival at the ED, he was reviewed by the stroke team at 17:20H. They found him confused during their assessment. Cranial nerves were grossly intact. His power in the four limbs was 5/5, and his sensation was grossly intact. His blood pressure on assessment was 164/82 mmHg, and his heart rate was 69 beats/min. Blood glucose on arrival was 6.2 mmol/L. His National Institutes of Health Stroke Scale (NIHSS) was 6. This score includes 2 points for aphasia, 2 points for the inability to follow commands, and 2 points for answering orientation questions. 

**Figure 1:**
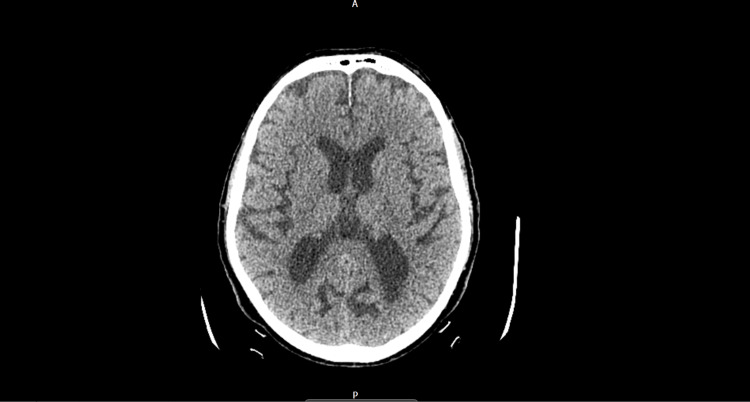
Non-contrast CT head. Non-contrast CT head of the patient done during the admission did not reveal any acute intracranial abnormalities/stroke or bleeding. CT, computed tomography

**Figure 2:**
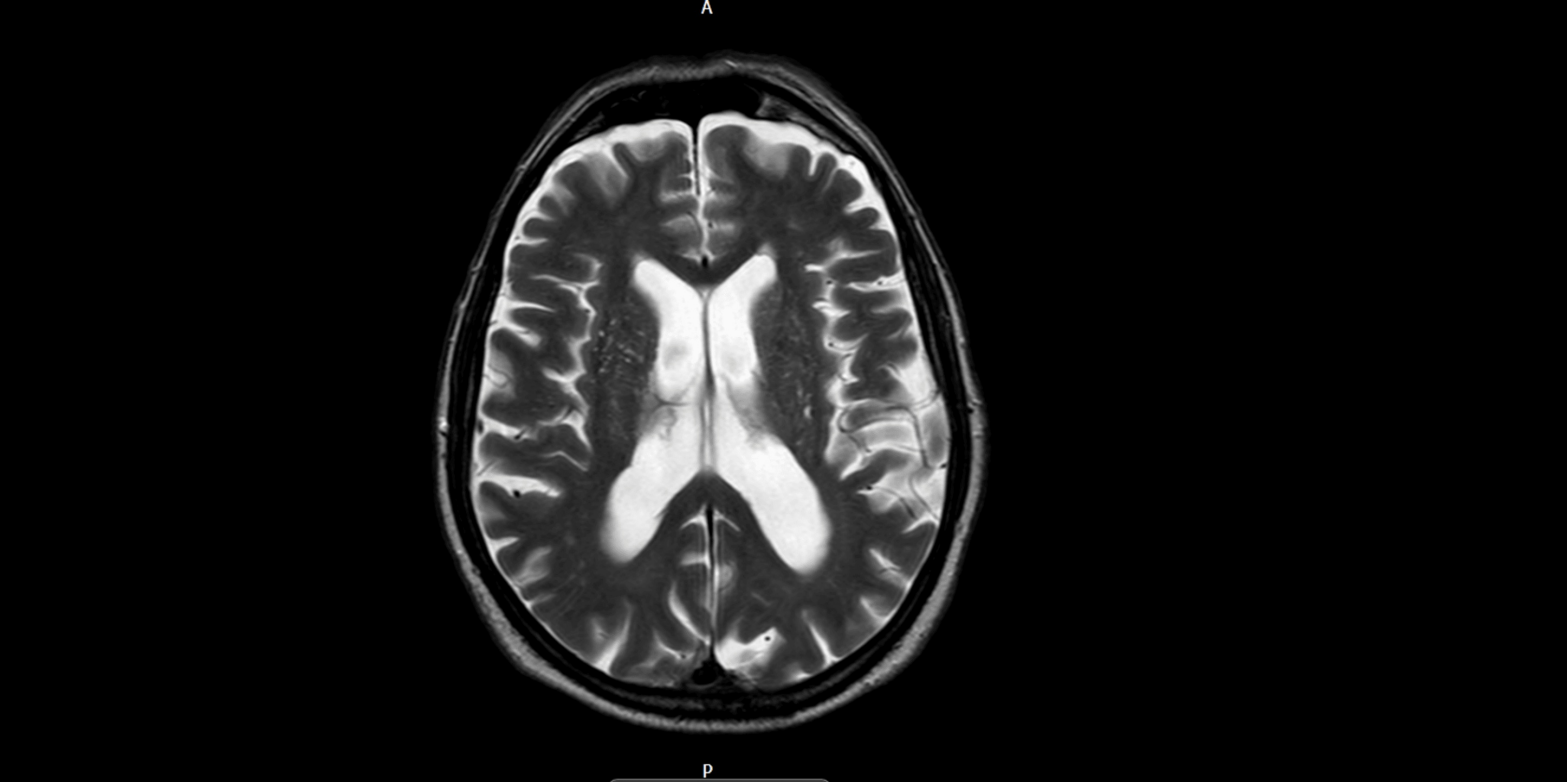
MRI head with contrast (axial T2 view). MRI head (axial T2 view): No acute infarct, hemorrhage, or mass lesion. Normal intracranial vascular flow voids. A small mature cortical infarct is seen in the left middle frontal gyrus on a background of mild chronic small vessel ischemic changes. Foci of microhemorrhage in the right frontal white matter and left occipital lobe are unchanged from previous imaging. The patient has previously been labeled as having multiple transient ischemic attacks during earlier admissions.

**Figure 3:**
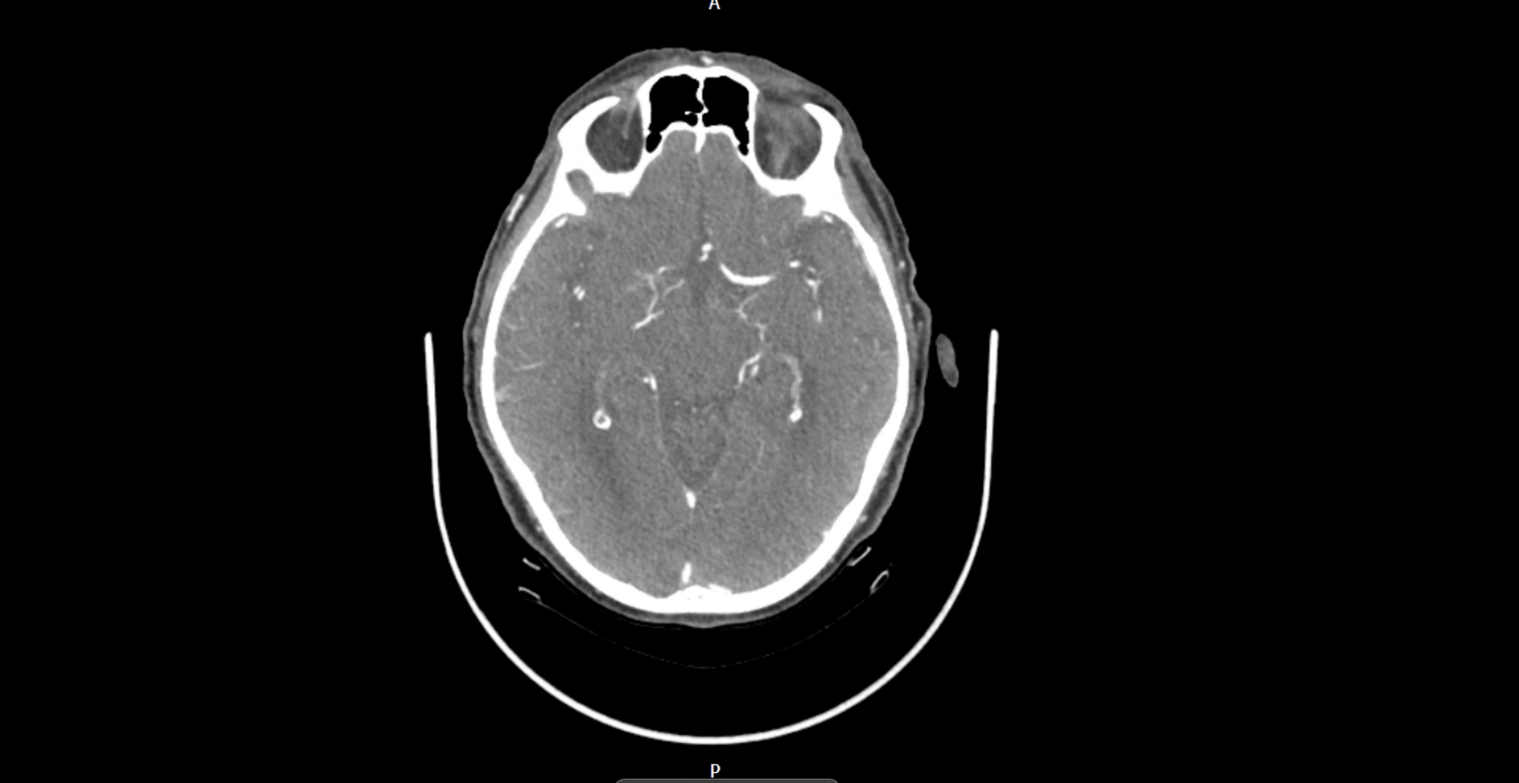
CT angiogram (intracranial, bilateral carotids, and aortic arch). Good opacification of the bilateral common carotid arteries. Mild to moderate narrowing of the bilateral carotid bulbs and proximal internal carotid arteries, with good opacification of the distal internal carotid arteries. Azygous anterior cerebral artery with good opacification of the distal segments. Good opacification of the bilateral middle cerebral arteries. Mildly hypoplastic left vertebral artery with a non-opacified distal intracranial segment. Good opacification of the right vertebral artery, basilar artery, and bilateral posterior cerebral arteries. No acute intracranial abnormalities detected; no evidence of thrombosis in the major intracranial arteries. CT, computed tomography

A stroke pathway was initiated for his admission. They performed a computed tomography (CT) of the brain, CT angiograms of the intracranial vessels, bilateral carotids, and aortic arch, as well as a magnetic resonance imaging (MRI) of the brain (Figure 1, Figure 2, and Figure 3, respectively). The reports mentioned moderate age-related small-vessel ischemic changes and mild generalized cerebral volume loss. No other acute intracranial evidence of thrombosis of the major intracranial arteries was identified. An MRI of the brain showed a small mature infarct with microhemorrhages in the right frontal and occipital lobes. We decided that he was not a candidate for thrombolysis because he had received anticoagulation treatment on the day of the incident after discussing it with the stroke consultant. He was then admitted to a geriatric ward, and his symptoms resolved spontaneously. His blood results during admission are shown in Table 1. Due to multiple recurrent admissions for similar symptoms, we performed a thorough review of his previous admissions and imaging (Table 2).

**Table 1:** Patient's blood investigation upon admission. *Abnormal results HDL, high-density lipoproteins; LDL, low-density lipoproteins; TSH, thyroid-stimulating hormone; FT4, free thyroxine 4; HbA1C, glycated hemoglobin

Blood investigation	Results	Range	Units
Hemoglobin	117*	130-180	g/L
White blood cell	7.1	4.0-11.0	10×9/L
Platelets	227	150-400	10×9/L
Sodium	135	133-146	mmol/L
Potassium	4.4	3.5-5.3	mmol/L
Urea	6.8	2.5-7.8	mmol/L
Creatinine	88	59-107	umol/L
HbA1C	41	20-41	mmol/mol
Cholesterol	3.5		mmol/L
HDL cholesterol	1.5		mmol/L
Triglyceride	0.88	<2.26	mmol/L
LDL cholesterol	1.6	<3	mmol/L
Magnesium	0.71	0.7-1.0	mmol/L
Phosphate	0.87	0.8-1.5	mmol/L
TSH	0.63	0.3-5.0	mU/L
FT4	11.3	7.9-16	pmol/L
Urine dip	No proteinuria, hematuria		

**Table 2:** Patient's previous admissions. N/A, not available; DOAC, direct oral anticoagulants; CT, computed tomography; MRI, magnetic resonance imaging; ECHO, echocardiogram; TIA, transient ischemic attack

Date	Symptoms	NIHSS	Thrombolysis	CT brain	CT angiogram (intracranial, carotids)	MRI head with contrast	Remarks/other investigations	Diagnosis
2-8 Aug 24	Sudden onset of expressive and receptive dysphasia and acute confusion	7	Thrombolysis after discussion with stroke consultant	No intracranial hemorrhage or acute infarction (pre- and post-thrombolysis)	No evidence of large or medium vessel occlusions	Moderate degree of cerebral atrophy. No microhemorrhages	Outpatient ECHO and Holter arranged	TIA
18-24 Sep 24	Confusion and dysphasia	0	N/A	No intracranial hemorrhage or acute infarct	N/A	N/A	N/A	Confusion and dysphasia for investigation
4-6 Oct24	Confusion and dysphasia	N/A	N/A	No acute intracranial hemorrhage or large territory infarct	N/A	No recent ischemia. Few white matter hyperintense foci on T2/T2 FLAIR suggestive of chronic small vessel ischemic changes. Small foci of old bleed in left occipital lobe	Ultrasound Doppler and carotid: There are calcified atheromatous plaques in bilateral CCA and ICA, which produce <30% stenoses	TIA
14-22 Nov 24	Confusion and dysphasia	3	Thrombolysis after discussion with stroke consultant	No intracranial hemorrhage or acute infarction (pre- and post-thrombolysis)	N/A	No hemorrhage or microhemorrhage. Chronic small-vessel ischemic disease. No acute intracranial finding	Started on DOAC in view of newly diagnosed AF. ECHO: EF 50%, no RWMA/shunts, no significant valve abnormalities	Newly diagnosed atrial fibrillation/dysphasia unlikely stroke-related
15-20 Jan 25	Confusion and dysphasia	6	No thrombolysis (patient on DOAC)	No acute infarct	No evidence of large or medium vessel occlusions	No evidence of recent infarct. Single probable previous microhemorrhage in right frontal lobe	Doppler carotid artery: calcified plaques in bulb and ICA. No sonographic evidence of significant stenosis	TIA
14 Feb 25	Slurred speech and right side weakness	N/A	N/A	No acute infarct	N/A	N/A	72 hours Holter monitoring: dominant rhythm is sinus with sinus arrhythmia. Frequent (1%) ventricular ectopics. No heart block seen.	TIA

During most of his admissions, he typically presented with an acute onset of confusion and dysphasia, which resolved spontaneously upon admission. His other imaging did show evidence of chronic small vessel ischemic changes and micro-hemorrhages; however, there was no evidence of acute infarction. An echocardiogram did not show evidence of shunting, and a CT angiogram of the intracranial, carotid, and aortic arch did not show evidence of large vessel occlusion. Holter monitoring was unremarkable as well. His case had been discussed with the stroke team, and they think that his presentation was related to a transient ischemic attack.

**Figure 4:**
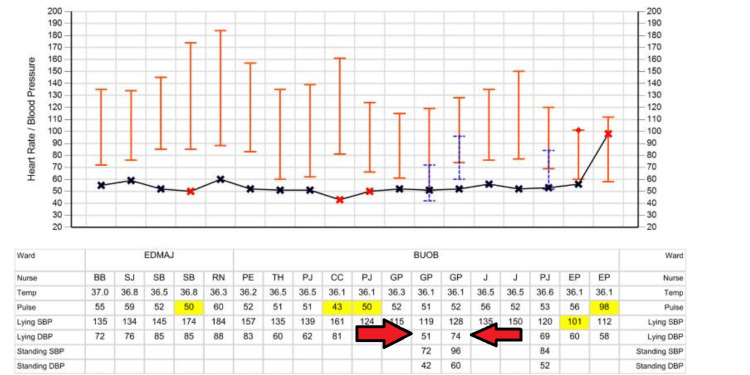
Clinical observation of the patient. Observation shows a significant lying/standing blood pressure drop, with a 47 mmHg systolic and 32 mmHg diastolic decrease. Temp, temperature (^o^C); pulse: heart rate (beats per minute); lying SBP, lying systolic blood pressure (mmHg); lying DBP, lying diastolic blood pressure (mmHg); standing SBP, standing (1 minute) systolic blood pressure (mmHg); standing DBP, standing (1 minute) diastolic blood pressure (mmHg)

Upon further discussion with the patient himself, he reported that he felt confused and had "word-finding difficulty" each time he finished walking his dog after a period of time. This condition also happens when he is exposed to a warm environment. Unlike what his wife described, his symptoms had never happened suddenly, and they resolved spontaneously within minutes. Otherwise, he denied any weakness, slurred speech, or other neurological deficits. These episodes, occurring post-exertion and resolving quickly, raised suspicion for cerebral hypoperfusion. We therefore performed a lying and standing blood pressure test, which confirmed the diagnosis of postural hypotension (Figure 4).

**Figure 5:**
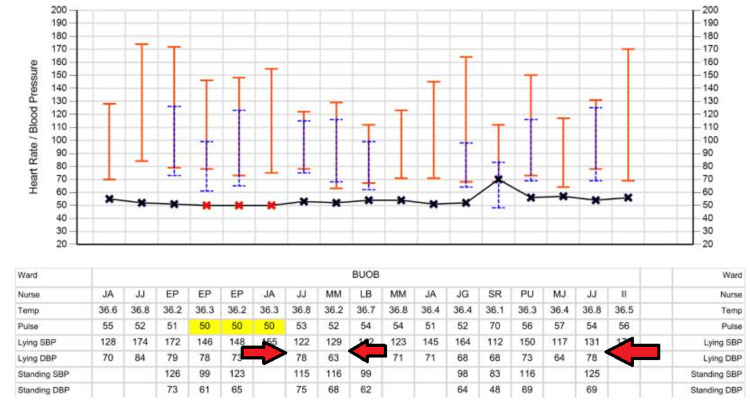
Clinical observation after management of postural hypotension. The diagram shows improvement of lying/standing blood pressure after pharmacological management of postural hypotension (<20 mmHg drop). Temp, temperature (^o^C); pulse: heart rate (beats per minute); lying SBP, lying systolic blood pressure (mmHg); lying DBP, lying diastolic blood pressure (mmHg); standing SBP, standing (1 minute) systolic blood pressure (mmHg); standing DBP, standing (1 minute) diastolic blood pressure (mmHg)

With the help of our physiotherapist, we prescribed him a set of lower limb exercises and thromboembolism-deterrent (TED) stockings to improve his postural hypotension. However, the postural blood pressure drop was still evident. We then started him on fludrocortisone 200 mcg once a day, which resulted in clinical improvement. After a few days, we discharged him in a stable condition (Figure 5). A retrospective review revealed no official documentation of lying or standing blood pressure measurements during any of his previous hospital admissions. He then continued his follow-up with the neurology clinic.

## Discussion

Postural hypotension is a common yet significant clinical condition among elderly patients. It is characterized by a significant reduction in blood pressure that typically occurs upon standing or assuming an upright posture [5,6]. In addition, it shares a close relationship with other prevalent chronic conditions such as hypertension, congestive heart failure, and Parkinson's disease. It is also associated with an increased risk of falls, cognitive impairment, and death, as well as reduced quality of life [7]. Moreover, it is an important differential diagnosis that may mimic an acute stroke event, as illustrated in this case report.

The initial management of postural hypotension should include specific interventions and guidelines, particularly a non-pharmacological approach. With the help of a physiotherapist, the patient should be fitted with TED stockings, maintain good hydration, and perform specific lower limb exercises. However, if the postural blood pressure drop is too significant or the patient remains symptomatic despite these measures, pharmacological treatment should be initiated with medications such as fludrocortisone or midodrine [8,9].

In this case study, the patient presented with a constellation of symptoms, presumably acute confusion and dysphasia, that resolved completely. Given imaging findings of chronic ischemic changes, cerebral vascular accident and transient ischemic attack should be the top differential diagnoses [10]. Regarding his cerebral microhemorrhages, this could be explained by the initiation of anticoagulants, as this finding was only evident after the patient began anticoagulation [11]. However, we must remain vigilant in broadening our differential diagnosis, especially in frail, elderly populations who present repeatedly with similar symptoms without a clear etiology. In this case, a thorough and concise history helped uncover another common yet underappreciated stroke mimic: postural hypotension.

## Conclusions

A comprehensive clinical history is necessary for an accurate diagnosis and suitable treatment because clinical presentations in elderly and frail patients can be unusual and easily missed. Simple bedside measurements like lying and standing blood pressure readings are crucial because conditions like postural hypotension can mimic more serious conditions like stroke. In appropriate situations, routine screening for postural hypotension can help prevent needless tests, treatments, and hospital stays.
